# Numerical Cognition in Neurodegenerative Disorders: A Systematic Review of Patterns of Preservation and Impairment

**DOI:** 10.3390/brainsci16101067

**Published:** 2026-10-04

**Authors:** Joël Macoir

**Affiliations:** 1 Faculté de Médecine, École des Sciences de la Réadaptation, Université Laval, Québec, QC G1V 0A6, Canada; joel.macoir@fmed.ulaval.ca; 2 Centre de Recherche CERVO—Brain Research Centre, Québec, QC G1J 2G3, Canada

**Keywords:** numerical cognition, number processing, calculation, acalculia, neurodegenerative diseases, neuropsychology

## Abstract

**Highlights:**

**What are the main findings?**

- Basic number comprehension and magnitude processing are often relatively preserved across neurodegenerative disorders, whereas numerical abilities requiring retrieval, transformation, or manipulation show greater vulnerability.
- Arithmetic fact knowledge shows variable preservation, while calculation procedures and complex calculation are among the most consistently affected numerical abilities.

**What are the implications of the main findings?**

- The dissociation between preserved and impaired numerical abilities supports a multicomponent organization of numerical cognition and highlights its differential vulnerability to neurodegenerative disease.
- Assessing multiple numerical domains may provide clinically relevant information beyond isolated calculation tasks, although current evidence remains insufficient to define reliable disease-specific numerical profiles.

**Abstract:**

Background/Objectives: Numerical cognition comprises multiple processes supporting the representation, comprehension, production, and manipulation of numerical information. Although numerical impairments have been described in neurodegenerative diseases, findings remain fragmented across disorders and numerical domains. This systematic review aimed to characterize patterns of preserved and impaired core numerical abilities across neurodegenerative disorders and to examine their implications for cognitive models and clinical assessment. Methods: PubMed, Web of Science, and PsycINFO were systematically searched for original empirical studies in English or French examining core numerical cognition in adults with neurodegenerative disorders or mild cognitive impairment. Reference lists were additionally screened. Methodological quality was appraised using design-specific Joanna Briggs Institute critical appraisal tools. Forty-seven studies published between 1989 and 2026 met the eligibility criteria. Findings were synthesized qualitatively because of substantial heterogeneity in study designs, clinical populations, and numerical measures. Results: Numerical profiles varied considerably across disorders, individuals, and disease stages, but several broad patterns emerged. Basic number comprehension and magnitude processing were often relatively preserved, whereas greater vulnerability was observed for processes requiring transformation, retrieval, or manipulation of numerical information. Arithmetic fact knowledge showed variable preservation, including dissociations between stored knowledge and its retrieval. Calculation procedures and complex calculation were among the most consistently affected abilities. Evidence was strongest for Alzheimer’s disease and semantic variant primary progressive aphasia and remained limited for several other neurodegenerative disorders. Conclusions: Numerical cognition is differentially affected across neurodegenerative diseases, supporting its multicomponent organization. These findings suggest the potential value of multidomain assessment rather than reliance on isolated calculation tasks. However, methodological heterogeneity, variation in methodological strength across studies, and the limited evidence available for several disorders reduce confidence in the generalizability of these patterns and currently preclude defining reliable disease-specific numerical signatures. Registration: OSF, 10.17605/OSF.IO/DU2Y9. Funding: None.

## 1. Introduction

Numbers are ubiquitous in everyday life. Numerical abilities are required for activities such as managing finances, shopping, interpreting dates and times, following medication instructions, and dealing with quantities and measurements. Consequently, acquired impairments of number processing and calculation can interfere substantially with everyday functioning and independence [1]. Although difficulties with numbers may manifest most visibly in complex everyday activities, functional numeracy should be distinguished from the core cognitive mechanisms that support numerical processing. Whereas functional numeracy concerns the effective use of numerical information in everyday contexts, core numerical cognition encompasses the representations and processes involved in understanding, producing, retrieving, and manipulating numerical information [2]. The present review focuses primarily on symbolic numerical processing, including number comprehension and production, transcoding, arithmetic fact knowledge, and calculation.

Numerical cognition is not a unitary ability but comprises partially dissociable representational and processing components. In the cognitive neuropsychological model proposed by McCloskey and colleagues, number processing involves distinct comprehension and production mechanisms, together with separate systems for arithmetic facts and calculation procedures [3]. The triple-code model provides a complementary account in which numbers can be represented in three principal formats: an analog magnitude code representing numerical quantity, a visual-Arabic code representing numerical symbols, and an auditory-verbal code supporting verbally mediated numerical processing [4,5]. Despite important differences between these models, both emphasize the multicomponent nature of numerical cognition and provide a framework for understanding selective patterns of preserved and impaired numerical abilities following brain dysfunction. Accordingly, the present review considers four broad domains: number comprehension, magnitude, and numerical semantics; number production and transcoding; arithmetic facts and arithmetic knowledge; and calculation procedures and complex calculation.

Numerical cognition is particularly relevant in the context of neurodegenerative disease. Most research has focused on Alzheimer’s disease (AD) and semantic variant primary progressive aphasia (svPPA), with a smaller body of evidence in mild cognitive impairment (MCI) and only limited investigation of posterior cortical atrophy (PCA), Parkinson’s disease (PD), logopenic variant primary progressive aphasia (lvPPA), and frontotemporal dementia (FTD). These disorders differ substantially in their predominant cognitive manifestations and patterns of neurodegeneration. Although the evidence available for some conditions remains sparse, their comparison provides an opportunity to examine whether different components of numerical cognition show differential vulnerability as language, semantic memory, visuospatial processing, memory, or executive functions deteriorate. Conversely, patterns of preservation and impairment across disorders may help clarify the extent to which particular numerical abilities depend on these broader cognitive systems. Neurodegenerative disorders thus provide a valuable framework for investigating both the clinical expression and the functional organization of numerical cognition.

Despite several decades of research, however, the literature remains fragmented. Studies have examined different neurodegenerative disorders using markedly heterogeneous approaches, ranging from comprehensive number-processing batteries to experimental tasks targeting specific abilities such as magnitude processing, transcoding, arithmetic fact retrieval, or calculation. The evidence is also distributed unevenly across disorders and numerical domains and includes both group investigations and detailed single-case studies. This diversity makes it difficult to determine whether consistent patterns of preservation and impairment emerge within and across neurodegenerative conditions. Previous reviews have addressed the cognitive and neural organization of numerical cognition and acquired acalculia more generally (e.g., [6,7]), whereas others have focused on numerical abilities in specific neurodegenerative conditions (e.g., [8]), including an earlier critical review of dementia that focused primarily on AD [9]. To our knowledge, however, the literature has not been systematically synthesized across neurodegenerative disorders and the major domains of core numerical cognition.

The present systematic review therefore aimed to synthesize the available evidence on core numerical cognition across neurodegenerative disorders. In the present review, core numerical cognition was defined as the cognitive processes supporting the representation, comprehension, production, manipulation, and calculation of numerical information, with primary emphasis on symbolic numerical processing. Specifically, we sought to (1) characterize patterns of preserved and impaired numerical abilities across disorders; (2) examine these findings across major components of numerical cognition, including number comprehension, magnitude, and numerical semantics; number production and transcoding; arithmetic facts and arithmetic knowledge; and calculation procedures and complex calculation; and (3) consider their implications for cognitive and neuroanatomical accounts of numerical cognition and for clinical assessment. By integrating findings across disorders and numerical domains, this review seeks to clarify which components of numerical cognition appear relatively resilient or vulnerable to neurodegenerative disease and to identify the main gaps in the current evidence.

## 2. Materials and Methods

### 2.1. Search Strategy

A systematic literature search was conducted to identify studies examining number processing in neurodegenerative disorders. The search strategy was developed to capture both clinical diagnostic groups and key domains of numerical cognition. Three electronic databases were searched: PubMed, PsycINFO (via Ovid), and Web of Science Core Collection. In addition to the database searches, the reference lists of included articles were manually screened to identify additional eligible studies. No other supplementary search sources were used.

Searches combined controlled vocabulary terms (e.g., MeSH or database-specific subject headings, when available) and free-text keywords related to neurodegenerative disorders and numerical cognition. Diagnostic terms included *Alzheimer’s disease*, *dementia*, *mild cognitive impairment*, *primary progressive aphasia*, *frontotemporal dementia*, *Parkinson’s disease*, *Lewy body disease*, and *posterior cortical atrophy*. Numerical cognition terms included *acalculia*, *dyscalculia*, *number processing*, *numerical cognition*, *number comparison*, *magnitude comparison*, *number transcoding*, *number line*, *mental number line*, *numerical magnitude*, and related terms. Search strategies were adapted to the syntax and indexing structure of each database.

No publication-date restrictions were applied. No language filters were applied in the three databases; eligibility was instead restricted to articles published in English or French during screening. The initial searches of PubMed, PsycINFO (Ovid), and Web of Science Core Collection were conducted on 20 April 2026. The same database-specific search strategies were rerun on 16 July 2026, with no publication-date restrictions, to identify potentially eligible records added since the initial searches. The complete database-specific search strategies, reconstructed retrospectively from the author’s search records, are provided in Supplementary Material S2; minor differences in syntax or formatting from the exact queries originally executed cannot be excluded.

All retrieved records were exported and imported into Covidence for reference management, duplicate removal, and screening.

### 2.2. Study Selection and Data Extraction

The review was reported in accordance with the Preferred Reporting Items for Systematic Reviews and Meta-Analyses (PRISMA 2020) statement [10]. This systematic review was retrospectively registered with the Open Science Framework (OSF) on 24 August 2026 (https://doi.org/10.17605/OSF.IO/DU2Y9). The registration was completed after the review and manuscript had been finalized and should therefore not be considered a prospective preregistration. After automatic and manual removal of duplicate records, titles and abstracts were screened for potential eligibility. Full texts were subsequently retrieved and assessed for all potentially eligible studies. Title/abstract screening, full-text eligibility assessment, and data extraction were conducted by the author.

Studies were eligible for inclusion if they: (1) reported original empirical data; (2) were written in English or French; (3) included adults with a clinically diagnosed neurodegenerative disorder or mild cognitive impairment associated with a neurodegenerative disease; and (4) assessed at least one component of core numerical cognition using a standardized or experimental numerical task. A neurodegenerative disorder was considered clinically diagnosed when the original study reported a clinical diagnosis established by the treating or research team. Explicit reporting of formal diagnostic criteria was not required for inclusion. For MCI samples, the presumed neurodegenerative etiology was based on the diagnostic characterization provided by the original study and was not independently established for this review. Eligible numerical domains included number comprehension, number production, numerical transcoding, magnitude representation and processing, numerical comparison and ordering, arithmetic fact retrieval, and mental or written calculation. These domains were defined a priori, before study selection and data extraction, and subsequently guided the organization of data extraction and the qualitative synthesis.

For the purposes of the present review, core numerical cognition was defined as the cognitive processes supporting the representation, comprehension, production, manipulation, and calculation of symbolic numerical information. Functional numeracy (e.g., money management, financial transactions, time estimation, medication dosage, or other everyday numerical activities) was considered outside the primary scope of the review because these activities depend not only on numerical cognition but also on the interaction of executive functions, memory, language, and visuospatial abilities. Moreover, studies assessing only non-symbolic numerosity perception (e.g., visual enumeration or subitizing) without a broader assessment of symbolic numerical cognition were excluded, as the present review focused on the representation, comprehension, production, manipulation, and calculation of symbolic numerical information. Studies that included both measures of core numerical cognition and functional numeracy were retained; however, only data pertaining to core numerical cognition were extracted and synthesized. Similarly, studies including both eligible symbolic numerical measures and non-symbolic numerosity measures were retained, and findings from the latter were reported when relevant to the characterization of numerical cognition.

Studies were excluded if they: (1) focused on non-neurodegenerative populations (e.g., stroke, traumatic brain injury, psychiatric or developmental disorders); (2) did not include any direct assessment of core numerical cognition; (3) did not report sufficient methodological detail and outcome data to permit extraction and synthesis of at least one component of core numerical cognition; (4) used numerical stimuli or tasks primarily to investigate neural activation or other nonnumerical processes without providing an independent behavioral assessment of core numerical cognition; (5) included mixed diagnostic groups for which numerical cognition outcomes were not reported separately by diagnosis; (6) relied solely on numerical items embedded within global cognitive screening instruments (e.g., serial subtraction); (7) assessed exclusively functional numeracy without including any measure of core numerical cognition; or (8) were reviews, editorials, conference abstracts lacking sufficient methodological information, or other non-empirical publications.

For each included study, data were extracted on publication characteristics (authors, year, and country), study design, diagnostic group(s), sample characteristics, numerical tasks administered, and comparator groups when applicable. Information was also collected on the specific numerical domains assessed, the principal findings, the authors’ interpretations, the cognitive mechanisms proposed to account for numerical impairments (e.g., semantic memory, executive functions, working memory, or visuospatial processing), and the methodological strengths and limitations of each study. These data formed the basis of the qualitative synthesis presented in the Results section.

### 2.3. Methodological Appraisal

The methodological quality of all included studies was appraised using design-specific Joanna Briggs Institute (JBI) critical appraisal checklists. Analytical cross-sectional studies were assessed using the revised JBI Critical Appraisal Tool for the Assessment of Risk of Bias for Analytical Cross-Sectional Studies [11], individual single-case investigations using the JBI Checklist for Case Reports [12], case series and multiple-case investigations using the JBI Checklist for Case Series [13], and longitudinal cohort studies using the revised JBI Critical Appraisal Tool for the Assessment of Risk of Bias for Cohort Studies [14]. Each applicable item was rated as Yes, No, Unclear, or Not applicable. Because the checklists assess design-specific methodological features, no aggregate quality score or cross-design ranking was calculated. Study-level appraisal results are provided in Supplementary Material S1 and were considered when interpreting the strength and consistency of the evidence. The methodological appraisal was conducted by the author.

## 3. Results

### 3.1. Study Selection

The database searches identified 395 records: 135 from PubMed, 112 from PsycINFO and 148 from Web of Science Core Collection. After removal of 175 duplicate records, 220 unique records remained for title and abstract screening. Of these, 117 were excluded, leaving 103 reports for full-text assessment. An additional seven studies were identified through manual screening of the reference lists of included articles and were also assessed for eligibility. Following full-text review, 63 articles were excluded based on the predefined eligibility criteria. In total, 47 studies met all inclusion criteria and were included in the qualitative synthesis. The study selection process is illustrated in Figure 1.

### 3.2. Main Characteristics of the Studies

The final review included 47 studies published between 1989 and 2026, spanning nearly four decades of research on core numerical cognition in neurodegenerative disorders (Figure 1). Because several studies included more than one diagnostic group, the numbers reported below are not mutually exclusive. AD was by far the most extensively investigated condition, represented in 27 studies (e.g., [15,16,17,18,19,20]). Three studies included participants with AD whose performance on numerical processing tasks was compared with that of participants with other neurodegenerative conditions such as corticobasal degeneration and frontotemporal dementia [21] or frontotemporal dementia and semantic dementia [22,23]. Five studies included participants with MCI, either as a separate group [24,25] or together with patients with AD [26,27,28]. Twelve studies included patients with semantic variant primary progressive aphasia (svPPA) or semantic dementia (SD), either as the primary clinical population [29,30,31,32,33] or as a diagnostic subgroup in studies comparing multiple neurodegenerative conditions [22,23]. Because most of these studies predated current consensus terminology, studies using the historical diagnostic label semantic dementia (SD) were grouped under svPPA to harmonize terminology across studies. This approach reflects the substantial clinical and conceptual overlap between the two constructs and was not intended to retrospectively re-diagnose individual participants. However, because the original studies did not necessarily report all features required by contemporary diagnostic criteria, equivalence between historically defined SD and svPPA should be considered approximate rather than complete. Two studies investigated posterior cortical atrophy (PCA) [34,35], two investigated Parkinson’s disease (PD) [36,37], one investigated logopenic variant primary progressive aphasia (lvPPA) [38], and three included participants with frontotemporal dementia (FTD) [21,23]. In studies including more than one neurodegenerative condition, numerical cognition outcomes were reported separately by diagnostic group. The main characteristics of the included studies are summarized in Table 1.

The methodological characteristics of the included studies varied considerably. Of the 47 included studies, 29 used cross-sectional group designs, 14 were single-case studies, one used a multiple single-case design, two were case series, and one was a longitudinal group study. Five of the 14 single-case studies also included longitudinal follow-up. Early investigations were dominated by single-case and small case-series studies, particularly in AD, svPPA, and PCA, reflecting the historical use of cognitive neuropsychology to characterize selective numerical impairments and infer the functional architecture of numerical cognition. These detailed studies provided important insights into dissociations between preserved and impaired numerical abilities and played a central role in the development of theoretical models of numerical cognition. More recent research has increasingly adopted cross-sectional group designs, often combining comprehensive neuropsychological assessment with structural or functional neuroimaging to investigate the neural correlates of numerical deficits. Nevertheless, relatively few studies included moderate-to-large patient cohorts, and longitudinal investigations remained uncommon. Numerical assessment varied substantially, ranging from comprehensive batteries to tasks targeting individual domains such as transcoding, magnitude processing, arithmetic fact retrieval, or calculation.

The methodological appraisal indicated generally adequate diagnostic ascertainment and participant characterization across the included studies, although methodological rigor varied across designs (Supplementary Material S1). Among analytical cross-sectional studies, inclusion and diagnostic criteria were generally well specified, outcome assessment and statistical analyses were largely appropriate, and samples were adequately described. The most recurrent limitation concerned the management of potential confounding factors: although relevant demographic or cognitive variables were frequently identified and groups were often matched on key characteristics, several studies did not otherwise address potential confounding in their design or statistical analyses. Single-case studies generally provided detailed clinical, neuropsychological, and experimental characterization; items of the JBI Case Report checklist relating specifically to therapeutic interventions were not applicable to these observational cognitive-neuropsychological investigations. Limitations in the small number of case-series and longitudinal studies primarily concerned documentation of case inclusion or completeness of follow-up.

### 3.3. Numerical Cognition Across Neurodegenerative Conditions

Given the marked heterogeneity in the number and type of numerical domains investigated across studies, findings are synthesized below by neurodegenerative condition, with results organized by numerical domain within each condition when appropriate.

#### 3.3.1. Alzheimer’s Disease

Studies of AD revealed substantial heterogeneity in both the severity and nature of numerical impairment. Basic number comprehension and magnitude processing were relatively preserved in several studies despite poorer arithmetic performance [17,22,26,55], although broader impairment was also reported [15,16], and Roquet et al. [19] identified impaired numerosity judgments associated with inhibitory functioning. Martini et al. [52], conversely, found largely preserved conventional numerical abilities despite difficulties with more ecologically complex numerical tasks.

Transcoding difficulties were frequent but heterogeneous. Group studies documented substantial variation in error patterns [39,42,48], with cross-linguistic differences suggesting an influence of language and number-word structure [54]. Intrusion and perseverative errors were reported by Macoir et al. [18,27], whereas single-case studies demonstrated more selective impairments involving specific transcoding routes or Arabic numeral reading [40,41,47].

Arithmetic and calculation were among the most consistently affected domains. Performance generally worsened with disease severity [43,44,50], with left temporoparietal metabolic correlates reported by Hirono et al. [45]. More specific deficits involved calculation procedures [46], arithmetic fact retrieval [51], arithmetic rules despite preserved multiplication facts [53], and broader arithmetic impairment [21,23]. Conversely, arithmetic memory retrieval could remain preserved despite increased susceptibility to inhibitory demands [28], and substantial interindividual variability was observed despite hippocampal atrophy [56].

Longitudinal and single-case studies further demonstrated dissociations between numerical components, including relative preservation of quantity knowledge despite progressive deterioration elsewhere [20] and preserved number processing despite impaired arithmetic procedures [49].

#### 3.3.2. Mild Cognitive Impairment

Five studies examined MCI. Zamarian and Stadelmann et al. [25] and Zamarian and Semenza et al. [28] found preserved retrieval of arithmetic knowledge but poorer performance when tasks imposed greater inhibitory demands. Benavides-Varela et al. [24] reported broader difficulties involving number comprehension, transcoding, numerical rules, and written calculation, whereas Jurado and Rosselli [26] found relatively preserved elementary numerical representations but poorer rule-based arithmetic performance. Macoir et al. [27] additionally documented transcoding errors, including perseverations, lexical errors, and intrusions.

Overall, the limited and heterogeneous evidence available suggests that elementary numerical knowledge may be relatively preserved in MCI, with greater variability on tasks requiring transcoding, arithmetic rules, or executive control. However, the small number of studies precludes definition of a consistent MCI-specific profile.

### 3.4. Semantic Variant Primary Progressive Aphasia

Across the twelve svPPA studies, severe deterioration of general semantic knowledge did not necessarily entail comparable loss of numerical knowledge. Relatively preserved numerical abilities despite marked semantic impairment were documented across single-case and group studies [30,32,57,58,59], with Papagno et al. [61] similarly reporting preserved quantity-based number processing, transcoding, and calculation procedures despite advanced semantic deterioration. Although specific deficits in arithmetic facts, procedures, or mathematical symbols were sometimes present, Papagno et al. (2013) further showed that direct retrieval of multiplication facts could be impaired despite preserved recognition of the correct answers and effective use of compensatory calculation strategies. Longitudinally, Cappelletti et al. [30] documented progressive calculation and procedural difficulties, whereas Jefferies et al. [33] observed increasing impairment of verbal numeral processing and transcoding despite comparatively preserved nonverbal magnitude knowledge.

Considerable interindividual variability was nevertheless evident. Cappelletti et al. [22] found numerical and arithmetic knowledge to be better preserved than nonnumerical semantic knowledge, whereas Crutch and Warrington [23] reported marked impairment of quantity facts. Luzzi et al. [60] identified deficits in arithmetic-sign recognition, fact retrieval, and calculation in most patients, while Diesfeldt [31] showed impaired arithmetic fact retrieval despite preserved procedures. Papagno et al. (2013) adds an intermediate pattern, in which arithmetic-fact retrieval was selectively vulnerable while arithmetic principles and procedural calculation remained relatively intact.

Thus, svPPA is characterized by relative rather than universal preservation of numerical cognition, with marked variability across components, individuals, and disease stages.

### 3.5. Posterior Cortical Atrophy

PCA was examined in two studies. In a detailed single case, Delazer and Karner et al. [34] found heterogeneous number-processing impairment, with subtraction and division more affected than addition and multiplication. In a larger sample, Kas et al. [35] found acalculia in 25 of 39 patients (64%), with poorer calculation associated with reduced left angular gyrus perfusion. Thus, the limited available evidence indicates prominent but potentially heterogeneous calculation impairment in PCA.

### 3.6. Parkinson’s Disease

PD was investigated in only two studies, which differed substantially in the numerical abilities examined. Zamarian et al. [37] found that patients performed comparably to healthy controls on most measures of number processing and calculation, with difficulties becoming apparent mainly on more demanding mental calculation and calculation-span tasks. Thus, relatively elementary numerical abilities appeared largely preserved, whereas performance was more vulnerable when numerical processing imposed greater cognitive demands.

Loenneker et al. [36] extended the investigation of basic number processing by distinguishing patients with normal cognition (PD-NC) from those with level I mild cognitive impairment (PD-MCI). Performance of the PD-NC group was generally comparable to that of healthy controls, whereas differences were more apparent in PD-MCI, particularly for symbolic and non-symbolic magnitude processing. In contrast, overall transcoding accuracy did not distinguish PD-NC from PD-MCI, although the groups differed in the types of errors produced. Importantly, the observed magnitude-processing differences could not be fully accounted for by the domain-general cognitive measures considered in the analyses. Overall, the two studies suggest relative preservation of basic numerical processing in PD-NC, with vulnerability emerging for complex calculation and, in PD-MCI, magnitude processing.

#### 3.6.1. Logopenic Variant Primary Progressive Aphasia

LvPPA was investigated in a single study. Macoir et al. [38] compared 14 individuals with lvPPA with 28 demographically matched healthy controls using a comprehensive battery assessing number recognition and comprehension, number production/transcoding, and calculation. Numerical performance was reduced across all three domains, but the severity of impairment followed a clear gradient. Recognition and comprehension were least affected, transcoding showed an intermediate level of impairment, and calculation was the most severely compromised domain. At the task level, basic recognition of Arabic digits and number words and magnitude comparison remained relatively preserved, whereas difficulties were found for several transcoding tasks. Calculation deficits were broader, encompassing arithmetic symbol production, all four basic mental arithmetic operations, and word-problem solving.

Group differences in overall numerical performance remained significant after separately controlling for language performance and global cognitive status. Although based on a single study, the findings therefore suggest a graded pattern of impairment, from relatively preserved recognition/comprehension to greater transcoding and particularly calculation difficulties.

#### 3.6.2. Frontotemporal Dementia

Three studies included FTD subgroups. Crutch and Warrington [23] reported difficulties across quantity-related knowledge, arithmetic operations, and arithmetic facts, although the absence of a healthy control group limits interpretation. Cappelletti et al. [22] found impaired calculation with more variable performance across other numerical domains.

Halpern et al. [21] provided the clearest group-level evidence, showing poorer oral calculation than in healthy controls, particularly for division, together with an association between working memory and calculation accuracy. Overall, the available evidence points primarily to calculation vulnerability, but remains insufficient to define a characteristic FTD numerical profile.

## 4. Discussion

Taken together, these findings indicate substantial heterogeneity in numerical cognition across neurodegenerative disorders, both within and across diagnostic groups. To identify broader patterns of preservation and impairment, the following discussion considers the evidence across numerical domains before examining their implications for clinical assessment.

### 4.1. Number Comprehension, Magnitude, and Numerical Semantics

Across disorders, elementary numerical representations appear relatively resilient compared with operations requiring transformation or manipulation of numerical information. This dissociation is clearest in AD, where magnitude and basic number processing can remain intact despite arithmetic impairment, although broader numerical deficits also occur [15,16,17,22,26,55]. Thus, numerical impairment in AD need not reflect degradation of quantity representations themselves but may arise from impaired access to or manipulation of otherwise available numerical information.

SvPPA provides converging evidence for a distinction between numerical and nonnumerical semantic knowledge, although this interpretation should be tempered by the substantial contribution of single-case studies and small samples to this literature. Numerical magnitude and quantity knowledge can remain remarkably robust despite profound conceptual-semantic deterioration [22,30,32,57,58,59,61], although this dissociation is neither universal nor permanent [23,33].

Findings from other neurodegenerative conditions, although more limited, support a similar dissociation. In PD, elementary number processing was largely preserved in cognitively intact patients, whereas symbolic and non-symbolic magnitude difficulties became more apparent in patients with concomitant MCI [36]. In lvPPA, basic recognition and magnitude comparison were among the least affected numerical abilities despite substantial impairment in transcoding and calculation [38]. Thus, across diagnostically distinct conditions, elementary numerical representations often appear more resilient than processes requiring the transformation, retrieval, or manipulation of numerical information. However, the strength of this cross-disorder pattern varies considerably, as evidence for several conditions derives from only one or two studies and, in some instances, small clinical samples.

To interpret these behavioral findings in a broader neuroanatomical context, evidence from the neuroimaging literature may be informative. Magnitude representation has been strongly associated with bilateral intraparietal regions [62,63], whereas verbal, semantic, and executive aspects of numerical processing additionally recruit temporal, frontal, and language-related networks [64,65]. Consequently, neurodegenerative diseases may disrupt particular components of numerical cognition according to the distribution and progression of their underlying pathology rather than producing a generalized loss of number knowledge. The marked preservation of numerical concepts in some patients with svPPA is especially informative in this respect, whereas the emergence of magnitude-processing difficulties in cognitively impaired PD illustrates that even relatively elementary numerical representations may become vulnerable when degeneration extends beyond the networks primarily affected early in the disease.

### 4.2. Number Production and Transcoding

Number production and transcoding emerged as particularly vulnerable components of numerical cognition, especially in AD, but the nature of the impairment varied markedly across studies. In AD, errors were observed when converting between Arabic, spoken, and written verbal number formats, with lexical, syntactic, intrusion, and perseverative errors reported across both group and single-case investigations [18,27,39,41,42,47,48]. This heterogeneity is theoretically informative because transcoding requires several partly separable operations: identification of the input notation, activation of the corresponding lexical and syntactic representations, conversion into the target code, and selection and production of the appropriate output [66,67]. Consequently, similar behavioral failures may arise from disruption at different stages of processing rather than reflecting a unitary impairment of number transcoding.

Several findings further indicate that transcoding difficulties cannot be explained solely by degradation of numerical representations. The intrusion and perseverative errors observed in AD have been linked to failures of attentional control and inhibition, whereby elements of the source notation are insufficiently suppressed during production [18]. Such errors were reproduced in nonnumerical cross-case transcoding by the same patient, supporting a contribution from domain-general control mechanisms rather than a purely numerical impairment. Linguistic structure also appears to influence performance. Ting et al. [54] found substantially more intrusion errors in Chinese- than English-speaking patients with AD, suggesting that the organization of the language and number-word systems constrains the manifestation of transcoding impairment. Thus, number production may be especially sensitive to the interaction between numerical representations and linguistic and executive processes.

Evidence from other neurodegenerative conditions reinforces the distinction between the representation of numerical quantity and the ability to express that representation in a particular symbolic format. In late-stage svPPA, Jefferies et al. [33] observed greater deterioration for verbal numbers than for Arabic numerals, while nonverbal magnitude knowledge remained comparatively preserved. The patient could still relate Arabic numerals to quantities despite difficulty producing them in response to spoken number words or dot arrays. In lvPPA, transcoding was also substantially impaired, but less severely than calculation and more severely than basic number recognition and comprehension [38]. This graded pattern is consistent with the additional phonological, working-memory, and sequencing demands involved in transforming one numerical notation into another. By contrast, the relatively preserved transcoding accuracy reported in PD, including PD-MCI, suggests that this component is not uniformly vulnerable across neurodegenerative syndromes, although qualitative changes in error type may precede clear loss of accuracy [36].

Beyond the behavioral findings synthesized in this review, neurocognitive models suggest that transcoding is unlikely to depend on a single specialized cortical region. Contemporary models distinguish at least partially separable visual-Arabic, verbal, and magnitude representations. Within the influential triple-code framework [5], visual processing of Arabic numerals is associated with ventral occipitotemporal regions, whereas verbal number processing recruits predominantly left-hemisphere language networks [68]. Transcoding therefore requires coordinated interactions between distinct representational systems, together with executive mechanisms involved in selecting the target code and suppressing competing representations. This distributed architecture provides a plausible account of why transcoding errors can arise in disorders with quite different patterns of degeneration.

The contrast between svPPA and lvPPA is particularly informative in this regard. In svPPA, progressive degradation of the anterior temporal semantic system may weaken the mappings between verbal number forms, Arabic symbols, and their corresponding magnitudes, while parietally mediated magnitude representations remain relatively preserved. This account is consistent with the preferential vulnerability of verbal number processing observed in advanced disease [33,58]. In lvPPA, by contrast, neurodegeneration predominantly affects the left temporoparietal junction and inferior parietal regions, including the supramarginal gyrus, which are critically involved in phonological processing and verbal working memory [69,70]. Disruption of these language-related networks may therefore contribute to the difficulties in mapping between verbal and symbolic numerical representations during transcoding. In AD, where posterior temporoparietal regions are commonly affected and executive dysfunction may also emerge [71], the diversity of lexical, syntactic, intrusion, and perseverative errors is consistent with disruption occurring at several points within this distributed transcoding system.

### 4.3. Arithmetic Facts and Arithmetic Knowledge

Arithmetic facts constitute a particularly informative component of numerical cognition because they depend on long-established knowledge that can often be retrieved directly from memory, rather than requiring the execution of a calculation procedure [72]. The reviewed studies provide an opportunity to examine the extent to which such highly learned arithmetic knowledge remains accessible in the context of neurodegenerative disease. In AD, Duverne et al. [51] demonstrated impaired arithmetic fact retrieval despite relatively preserved ability to select strategies according to problem characteristics, suggesting that access to stored arithmetic knowledge can be impaired despite preservation of the capacity to adapt problem-solving behavior. Other studies revealed considerable heterogeneity: Allen et al. [53] reported preserved multiplication facts but impaired arithmetic rule retrieval, whereas Zamarian and Semenza et al. [28] found relatively preserved retrieval from arithmetic memory, with difficulties becoming more apparent when inhibitory demands increased. Arithmetic fact impairment in AD therefore appears neither obligatory nor readily attributable to a generalized loss of numerical knowledge. Rather, successful performance may depend on the integrity of both stored arithmetic associations and the cognitive mechanisms required to access them.

The findings in svPPA are especially informative for understanding the status of arithmetic knowledge within semantic memory. Several reports have described striking preservation of arithmetic facts despite severe deterioration of nonnumerical semantic knowledge, supporting at least partial functional independence between these knowledge systems [29,57]. Other patients, however, showed impaired arithmetic fact retrieval despite preservation of calculation procedures [31]. Papagno et al. [61] reported a similarly selective difficulty in retrieving multiplication facts, while recognition of the correct solutions remained intact and alternative calculation strategies could be used successfully. In a longitudinal study, Julien et al. [58] found that arithmetic performance worsened with the severity of semantic impairment; patients increasingly relied on basic and inflexible strategies to solve multiplication problems, challenging the notion that arithmetic knowledge is entirely independent of the semantic system. Taken together, these findings suggest relative and potentially long-lasting resilience of arithmetic knowledge rather than complete independence from semantic memory. This interpretation remains provisional, however, because much of the detailed evidence derives from single-case investigations and small samples.

This distinction may also help reconcile apparently contradictory findings across neurodegenerative disorders. Possessing arithmetic fact knowledge and successfully retrieving the corresponding answer are not necessarily equivalent. Performance may reflect the integrity of the stored association itself, access to its verbal representation, selection among competing answers, or use of compensatory calculation strategies when direct retrieval fails. Classical neuropsychological models have accordingly distinguished stored arithmetic facts from arithmetic rules and procedures [73]. The qualitative analyses in svPPA are particularly revealing in this respect: deterioration may be manifested not simply as incorrect answers but as a progressive shift away from automatic fact retrieval toward slower and less flexible strategies. Consequently, accuracy scores alone may underestimate changes in arithmetic knowledge during neurodegeneration.

From a neuroanatomical perspective, arithmetic fact retrieval appears to rely on a distributed, predominantly left-lateralized network rather than on a single specialized region. The left angular gyrus has nevertheless been consistently associated with retrieval-based arithmetic: its activity is greater when arithmetic problems are solved through direct fact retrieval than through procedural strategies [74], a distinction recently confirmed by meta-analytic evidence [75]. Arithmetic fact learning and retrieval also engage medial temporal structures, including the hippocampal system, suggesting that successful retrieval depends on interactions among parietal, temporal, and memory-related regions [76]. Arithmetic fact retrieval is therefore best conceived as relying on a distributed network, with the left angular gyrus particularly involved in accessing highly learned arithmetic associations.

### 4.4. Calculation Procedures and Complex Calculation

Calculation appears to be one of the most consistently vulnerable components of numerical cognition across neurodegenerative disorders. Unlike elementary number processing or direct retrieval of arithmetic facts, complex calculation requires the coordinated use of multiple processes, including knowledge of arithmetic operations and procedures, maintenance and manipulation of intermediate results, sequencing of calculation steps, and executive monitoring [77]. This multicomponent nature may help explain why calculation difficulties occur across disorders with otherwise markedly different cognitive profiles.

The AD literature illustrates how calculation impairment may reflect increasing procedural and executive demands rather than loss of elementary numerical knowledge. Performance declines with disease severity [43,44,50], complex operations may become affected before simpler ones [20,50], and both procedural execution and working-memory limitations contribute to performance [21,46]. Similar sensitivity to task complexity in FTD [21] and PD [37] further emphasizes the contribution of working memory and executive control to calculation.

Calculation may also be disproportionately affected when degeneration involves parietal numerical networks. Prominent calculation deficits in PCA, including an association with reduced left angular gyrus perfusion [35], and the marked calculation impairment observed in lvPPA [38] are consistent with involvement of inferior parietal and temporoparietal regions in these syndromes, although the small number of studies precludes a disease-specific neuroanatomical account [78,79].

The svPPA literature provides an informative contrast. Calculation and arithmetic procedures may remain remarkably preserved despite severe deterioration of nonnumerical semantic knowledge in some patients, whereas other studies documented progressive or substantial calculation impairment [29,30,31,57,59,60]. This variability reinforces the view that successful calculation cannot be reduced to the integrity of general semantic knowledge. Rather, its vulnerability depends on which numerical, linguistic, mnemonic, and executive components required by a particular calculation task are compromised.

From a neuroanatomical perspective, calculation relies on a distributed frontoparietal network rather than on a single arithmetic-specific region. Meta-analytic evidence indicates that calculation engages bilateral parietal regions involved in numerical processing together with prefrontal regions supporting the monitoring and manipulation of information [80]. Importantly, the contribution of these regions varies according to the strategy and cognitive demands of the task: procedural calculation engages a broader frontoparietal network, with preferential involvement of inferior frontal and cingulate regions, whereas direct arithmetic fact retrieval is more strongly associated with the left angular gyrus [75]. This distinction is consistent with the greater vulnerability of complex calculation observed across several neurodegenerative conditions, as increasing procedural, working-memory, and executive demands place greater reliance on distributed frontoparietal networks. More broadly, the findings suggest that calculation may be particularly vulnerable to neurodegeneration precisely because successful performance depends on the coordinated integrity of multiple numerical and domain-general cognitive systems.

### 4.5. Clinical Implications

From a clinical perspective, the present findings suggest potential value in assessing numerical cognition as a multidimensional construct rather than relying exclusively on calculation tasks or isolated numerical items embedded within global cognitive screening instruments [81]. Assessment across basic number comprehension and magnitude processing, transcoding, arithmetic fact knowledge, and calculation may reveal clinically meaningful dissociations between preserved and impaired abilities that would otherwise remain undetected. Such a multidomain approach may be informative because relatively elementary numerical abilities can remain preserved despite substantial impairment in more demanding numerical operations. However, the present review does not establish the diagnostic accuracy or prognostic value of multidomain numerical assessment, nor its superiority over existing clinical assessment approaches.

At the same time, translation of the available evidence into clinical practice remains challenging. Numerical cognition was assessed using highly heterogeneous instruments and experimental tasks, often differing in stimulus characteristics, response requirements, scoring procedures, and the cognitive demands imposed beyond numerical processing itself. Moreover, studies varied considerably in disease severity and cognitive status, making direct comparison of numerical profiles across studies and disorders difficult. The methodological appraisal further indicated that potential confounding factors were not consistently addressed across studies, which limits the extent to which some observed numerical differences can be attributed specifically to the neurodegenerative condition rather than to associated demographic or cognitive factors. This is particularly important in progressive conditions, because the pattern of preserved and impaired numerical abilities may change substantially over the course of the disease. Consequently, the profiles summarized in Table 2 should be viewed as qualitative clinical patterns rather than diagnostic signatures or disorder-specific benchmarks. Greater use of standardized, multidomain numerical batteries and clearer characterization of disease stage would facilitate comparison across neurodegenerative conditions and help determine the clinical utility of particular numerical measures.

### 4.6. Strengths and Limitations

Several strengths of this review should be acknowledged. Its focused definition of core numerical cognition allowed findings to be organized according to theoretically meaningful components rather than treating numerical ability as a unitary construct. The inclusion of a broad range of neurodegenerative conditions, together with both group and single-case studies, also allowed disease-specific patterns and informative dissociations between numerical abilities to be examined. Finally, organizing the Results by neurodegenerative condition and the Discussion by numerical domain provided complementary clinical and cognitive perspectives.

The available literature nevertheless presents important limitations. Numerical cognition was assessed using highly heterogeneous tasks and batteries, differing in complexity, response requirements, and associated cognitive demands. Clinical samples also varied substantially in disease severity, cognitive status, and diagnostic criteria, limiting comparisons across studies and disorders. The evidence base was markedly unbalanced across conditions, and many studies involved single cases or small samples, with relatively few longitudinal investigations. Numerical profiles should therefore be regarded as provisional, particularly for the less extensively studied disorders.

The present review also has limitations. Given the substantial heterogeneity of study designs, populations, measures, and outcomes, a quantitative meta-analysis was not considered appropriate and the synthesis remained qualitative. Diagnostic terminology and criteria also changed over the broad period covered by the review, particularly for semantic dementia, which was classified as svPPA. Despite searching three major databases and screening reference lists, relevant studies may have been missed because numerical cognition is sometimes examined as a secondary outcome and may not be clearly identified in titles, abstracts, or indexing terms. Restricting eligibility to studies published in English or French may have introduced language bias and resulted in the omission of relevant studies published in other languages. In addition, the grey literature was not systematically searched, which may have increased the risk of publication bias, particularly given the relatively small and historically dispersed literature in this field. Study selection, data extraction, and methodological appraisal were conducted by a single reviewer, which precluded independent verification and increased the possibility of selection, extraction, or appraisal errors. Given the heterogeneity of the included literature, this limitation reduces confidence in the completeness and accuracy of the review process. In addition, the retrospective registration of the review limited prospective methodological transparency, while the retrospective reconstruction of the database-specific search strategies from the author’s records limits exact reproducibility of the searches. These limitations should be considered when interpreting the consistency and robustness of the patterns identified in this review.

## 5. Conclusions

This systematic review suggests that numerical cognition is differentially affected across neurodegenerative disorders and cannot be adequately characterized as a unitary ability. Across the available literature, elementary number comprehension and magnitude representations often appear relatively resilient, whereas transcoding, arithmetic fact retrieval, and particularly complex calculation show greater vulnerability depending on the cognitive and neural systems affected by the disease. The marked dissociations observed both within and across neurodegenerative conditions further illustrate the multicomponent organization of numerical cognition and the contribution of language, semantic memory, working memory, and executive processes to numerical performance. Clinically, these findings suggest the potential value of assessing multiple numerical domains rather than relying on isolated calculation tasks or numerical items from general cognitive screening measures. However, substantial methodological heterogeneity and the limited evidence available for several disorders currently preclude defining reliable disease-specific numerical signatures. Future studies using standardized multidomain assessments, larger and better-characterized clinical samples, and longitudinal designs will be essential to determine the diagnostic and prognostic value of numerical cognition in neurodegenerative disease.

## Figures and Tables

**Figure 1:**
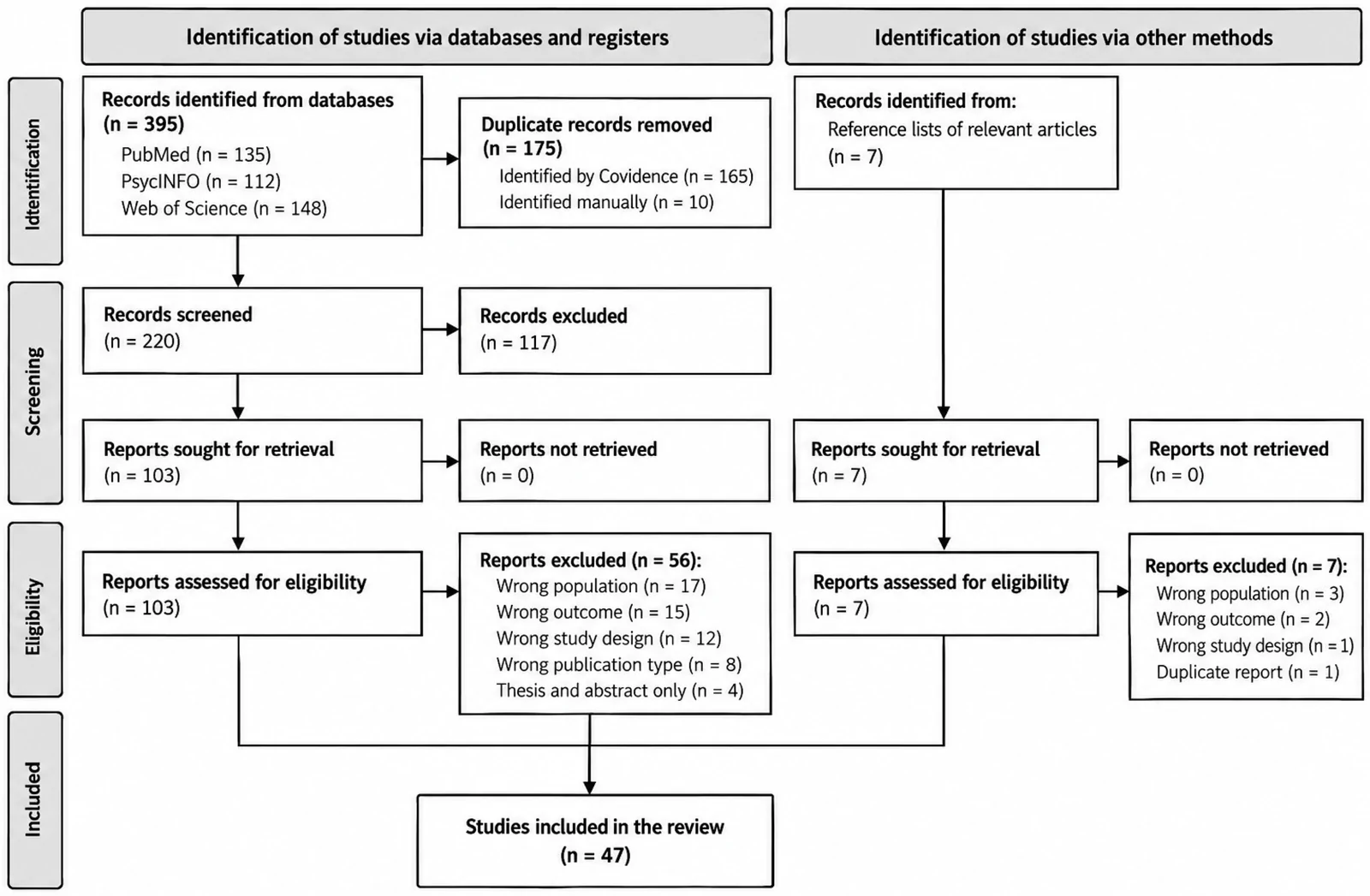
PRISMA flow diagram of the study selection process.

**Table 1:** Characteristics of the included studies.

Study (Year)	Clinical Sample (n)	Comparator Group(s)	Design	Numerical Domains Assessed	Key Findings
**Alzheimer’s disease**					
Grafman et al. (1989) [20]	1	None	Single-case (longitudinal)	Arithmetic facts; calculation	Progressive breakdown despite preserved numerosity knowledge
Tegnér & Nyback (1990) [39]	13	None	Cross-sectional	Numerical transcoding	Number transcoding impaired with intrusion errors
Cipolotti (1995) [40]	1	None	Single-case	Number comprehension; number production; numerical transcoding; calculation	Selective Arabic numeral reading dyslexia
Deloche et al. (1995) [16]	17	None; performance interpreted using standardized EC301 data	Cross-sectional	Number comprehension; number production; numerical transcoding; calculation	Heterogeneous impairment across numerical domains
Noël & Seron (1995) [41]	1	None; performance interpreted using standardized EC301 data	Single-case	Number comprehension; number production; numerical transcoding; calculation	Selective impairment of written verbal to Arabic transcoding
Kessler & Kalbe (1996) [42]	21	HC (n = 22)	Cross-sectional	Numerical transcoding	Number transcoding impaired with intrusion errors
Marterer et al. (1996) [43]	23	VD (n = 19); HC (n = 17)	Cross-sectional	Calculation	Severity-related decline in basic arithmetic
Rosselli et al. (1998) [44]	20	None	Cross-sectional	Mental calculation; written calculation	Severity-related decline in basic arithmetic
Hirono et al. (1998) [45]	91	None	Cross-sectional	Mental calculation; written calculation	Arithmetic impairment with left temporoparietal metabolic correlates
Mantovan et al. (1999) [46]	12	HC (n = 19); left-hemisphere lesion group (n = 10)	Cross-sectional	Number comprehension; number production; numerical transcoding; calculation	Impaired calculation particularly affecting the execution of arithmetic procedures
Thioux et al. (1999) [47]	1	None	Single-case (longitudinal)	Number comprehension; number production; numerical transcoding; calculation	Number transcoding impaired with intrusion errors
Carlomagno et al. (1999) [15]	68	HC (n = 242)	Cross-sectional	Number comprehension; number production; numerical transcoding; calculation	Global impairment across numerical domains
Della Sala et al. (2000) [48]	20	HC (n = 40)	Cross-sectional	Numerical transcoding	Heterogeneous transcoding impairment
Crutch & Warrington (2001) [23]	20 (AD subgroup)	FTD (n = 20); other progressive cortical conditions (n = 10)	Cross-sectional	Calculation	Impaired quantity facts, number operations, arithmetic facts, and calculation
Macoir et al. (2002) [18]	1	None; performance interpreted using standardized EC301 data	Single-case	Numerical transcoding	Number transcoding impaired with intrusion errors resulting from attentional deficit
McNeil & Burgess (2002) [49]	1	HC (n = 6)	Single-case	Number comprehension; number production; numerical transcoding; calculation	Preserved number processing except for arithmetical procedures
Kaufmann et al. (2002) [17]	19	HC (n = 19)	Longitudinal	Number comprehension; number production; numerical transcoding; calculation	Preservation of basic numerical representations with heterogeneous arithmetic impairment
Halpern et al. (2003) [21]	20 (AD subgroup)	CBD (n = 17); FTD (n = 17); HC (n = 17).	Cross-sectional	Mental calculation; dot counting	Impaired mental calculation
Martin et al. (2003) [50]	20	HC (n = 23)	Cross-sectional	Mental calculation; written calculation	Severity-related decline in calculation abilities
Duverne et al. (2003) [51]	16	Older HC (n = 16); younger HC (n = 16)	Cross-sectional	Magnitude processing; arithmetic facts	Impaired arithmetic facts retrieval with relatively preserved adaptive strategy selection
Martini et al. (2003) [52]	21	HC (n = 17)	Cross-sectional	Number comprehension; number production; numerical transcoding; calculation	Preservation of numerical cognition; deficits in ecologically complex tasks
Allen et al. (2005) [53]	18	Older HC (n = 20); younger HC (n = 20)	Cross-sectional	Arithmetic facts	Preservation of multiplication facts with impairment of rule retrieval
Zamarian, Semenza et al. (2007) [28]	15 (AD subgroup)	HC (n = 20)	Cross-sectional	Calculation	Preserved arithmetic memory retrieval with task-specific inhibitory-control difficulty
Cappelletti et al. (2012) [22]	8 (AD subgroup)	svPPA (n = 3); FTD (n = 3); nfPPA (n = 2); focal brain damage (n = 20); HC (n = 30)	Cross-sectional	Number comprehension; number production; numerical transcoding; calculation	Preserved quantity processing; impaired calculation and numerical transcoding
Ting et al. (2016) [54]	18	None	Cross-sectional	Numerical transcoding	Language-system-dependent transcoding impairment
Jurado & Rosselli (2017) [26]	20 (AD subgroup)	Older HC (n = 20); younger HC (n = 30)	Cross-sectional	Arithmetic facts; magnitude processing	Arithmetic-fact retrieval deficit with relatively preserved magnitude processing
Mendez et al. (2018) [55]	18	Other EOAD presentations (n = 258)	Retrospective case series	Magnitude processing; mental calculation; written calculation	Primary acalculia with preserved magnitude processing
Delazer et al. (2019) [56]	AD (n = 13); MCI (n = 3)	Age-matched normative data	Multiple single-case	Number comprehension; calculation	Heterogeneous impairment despite hippocampal atrophy
Roquet et al. (2020) [19]	25	HC (n = 23)	Cross-sectional	Non-symbolic dot comparison; symbolic number-line estimation	Impaired numerosity processing and inhibition
Macoir et al. (2020) [27]	8 (AD subgroup)	HC (n = 20)	Cross-sectional	Numerical transcoding	Number transcoding impaired with intrusion errors and perseverations
**Mild cognitive impairment**					
Zamarian, Stadelmann et al. (2007) [25]	18	Older HC (n = 18); younger HC (n = 18)	Cross-sectional	Calculation	Preservation of arithmetic memory retrieval with impairment of inhibitory processes
Zamarian, Semenza et al. (2007) [28]	18 (MCI subgroup)	HC (n = 20)	Cross-sectional	Calculation	Preserved arithmetic memory retrieval with task-specific inhibitory-control difficulty
Benavides-Varela et al. (2015) [24]	33	HC (n = 29)	Cross-sectional	Number comprehension; number production; numerical transcoding; calculation	Impaired number comprehension, numerical transcoding, numerical rules and written calculation
Jurado & Rosselli (2017) [26]	20 (MCI subgroup)	Older HC (n = 20); younger HC (n = 30)	Cross-sectional	Arithmetic facts; Magnitude processing	Preservation of basic numerical representations with impairment in rule-based operations
Macoir et al. (2020) [27]	20 (MCI subgroup)	HC (n = 20)	Cross-sectional	Numerical transcoding	Number transcoding impaired with perseverations, lexical and intrusion errors
**Semantic variant primary progressive aphasia**					
Diesfeldt (1993) [31]	1	HC (n = 6)	Single-case (longitudinal)	Numerical transcoding; mental calculation; written calculation	Impaired arithmetic facts retrieval with preserved arithmetical procedures
Cappelletti et al. (2001) [29]	1	HC (n = 7)	Single-case	Number comprehension; number production; numerical transcoding; calculation	Preserved numerical cognition with impaired knowledge of number facts and arithmetical procedures
Crutch & Warrington (2001) [23]	7 (svPPA subgroup)	AD (n = 20); FTD (n = 13); other progressive cortical conditions (n = 10)	Cross-sectional	Calculation	Marked impairment of quantity fact knowledge
Crutch & Warrington (2002) [32]	1	Age-matched HC (n = 1; problem-solving tasks only)	Single-case	Number comprehension; number production; numerical transcoding; calculation	Preservation of numerical cognition with impairment in understanding mathematical symbols
Cappelletti et al. (2005) [30]	1	Normative/control data (task-dependent)	Single-case (longitudinal)	Number comprehension; number production; numerical transcoding; calculation	Preservation of numerical cognition with progressive impairment of calculation and arithmetical procedures
Jefferies et al. (2005) [33]	1	Age- and education-matched HC (n = 12; digit-span task only)	Single-case	Numerical transcoding; magnitude processing	Impaired processing of verbal numerals; progressive impairment of transcoding; preserved knowledge of nonverbal number magnitude
Zamarian et al. (2006) [57]	1	HC (n = 15–18, depending on task)	Single-case	Number comprehension; number production; numerical transcoding; calculation	Complete preservation of numerical cognition
Julien et al. (2010) [58]	14	HC (n = 10)	Cross-sectional	Magnitude processing	Preservation of quantity knowledge
Cappelletti et al. (2012) [22]	3 (svPPA subgroup)	AD (n = 8); FTD (n = 3); nfPPA (n = 2); focal brain damage (n = 20); HC (n = 30)	Cross-sectional	Number comprehension; number production; numerical transcoding; calculation	Numerical and arithmetic knowledge relatively preserved despite severe semantic impairment
Cheng et al. (2013) [59]	2	HC (n = 6)	Case series	Number reading; magnitude and numerosity processing; counting; arithmetic; calculation	Numerical processing broadly preserved despite marked semantic impairment, including in the patient with more severe svPPA
Luzzi et al. (2013) [60]	8	HC (n = 9)	Cross-sectional	Calculation	In 6 of 8 patients, impaired arithmetic-sign recognition, arithmetic-fact retrieval, and calculation
Papagno et al. (2013) [61]	1	HC (n = 5–8, depending on task)	Single-case (longitudinal)	Number comprehension; number production; numerical transcoding; calculation	Preserved quantity-based number processing, transcoding, calculation procedures, and arithmetic principles; impaired retrieval of multiplication facts and nonquantitative number knowledge
**Posterior cortical atrophy**					
Delazer, Karner et al. (2006) [34]	1	HC (n = 5–40, depending on task); normative data for standardized tasks	Single-case	Number comprehension; number production; numerical transcoding; calculation	Generalized impairment of number processing
Kas et al. (2011) [35]	39	AD (n = 24); HC (n = 24)	Cross-sectional	Mental calculation	Impaired mental calculation
**Parkinson’s disease**					
Zamarian et al. (2006) [37]	15	HC (n = 28)	Cross-sectional	Number comprehension; number production; numerical transcoding; calculation	Impairment limited to complex mental calculation and calculation span tasks
Loenneker et al. (2024) [36]	PD-NC (n = 50); PD-MCI (n = 29)	HC (n = 40)	Cross-sectional	Numerical transcoding; magnitude processing; numerical comparison	Basic number processing largely preserved in PD-NC; PD-MCI showed impaired symbolic and non-symbolic magnitude processing, with relatively preserved transcoding
**Logopenic variant primary progressive aphasia**					
Macoir et al. (2026) [38]	14	HC (n = 28)	Cross-sectional	Number comprehension; number production; numerical transcoding; calculation	Generalized impairment of number processing with a graded pattern: calculation > transcoding > recognition/comprehension
**Frontotemporal dementia**					
Crutch & Warrington (2001) [23]	13 (FTD subgroup)	svPPA (n = 7); AD (n = 20); other progressive cortical conditions (n = 10)	Cross-sectional	Calculation	Better graded arithmetic than AD and better quantity-fact knowledge than svPPA
Halpern et al. (2003) [21]	17 (FTD subgroup)	AD (n = 20); CBD (n = 17); HC (n = 17)	Cross-sectional	Mental calculation; dot counting	Impaired oral calculation with relatively preserved numerosity; executive limitations likely contributed
Cappelletti et al. (2012) [22]	3 (FTD subgroup)	AD (n = 8); svPPA (n = 3); nfPPA (n = 2); focal brain damage (n = 20); HC (n = 30)	Cross-sectional	Number comprehension; number production; numerical transcoding; calculation	Preserved quantity processing despite impaired calculation and transcoding

Note. AD = Alzheimer’s disease; CBD = corticobasal degeneration; EOAD = early-onset Alzheimer’s disease; FTD = frontotemporal dementia; HC = healthy control; MCI = mild cognitive impairment; nfPPA = non-fluent variant primary progressive aphasia; PD-NC = Parkinson’s disease with normal cognition; PD-MCI = Parkinson’s disease with MCI; svPPA = semantic variant primary progressive aphasia; VD = vascular dementia. Studies including more than one neurodegenerative condition are listed under each relevant diagnostic category when numerical cognition outcomes were reported separately by diagnostic group.

**Table 2:** Summary of numerical cognition profiles across neurodegenerative disorders.

Condition	Evidence Base	Basic Number Comprehension/Magnitude	Transcoding	Arithmetic Facts Knowledge	Calculation
AD	27 studies; mixed designs, including group and single-case studies	Relatively preserved to impaired	Frequently affected; heterogeneous errors	Variable	Frequently affected; worsens with disease severity
MCI	5 studies; predominantly group studies	Generally preserved to mildly impaired	Impaired in some studies; heterogeneous errors	Generally preserved; rule-based processing may be vulnerable	Variable; difficulties increase with task demands
svPPA	12 studies; substantial contribution of single-case and small-sample studies	Often relatively preserved	Variable; verbal number processing may be particularly vulnerable	Variable, ranging from preserved to impaired	Variable
PCA	2 studies; one single case and one group study	Insufficient evidence	Insufficient evidence	Not specifically assessed	Impaired in both studies
PD	2 group studies	Relative preservation in PD-NC; magnitude difficulties reported in PD-MCI	Relative preservation in both studies	Not specifically assessed	Complex calculation particularly vulnerable
lvPPA	1 group study	Relatively preserved	Impaired	Impaired	Markedly impaired
FTD	3 studies; small clinical subgroups	Relatively preserved in available studies	Impaired in one study	Limited and variable evidence	Impaired; executive demands may contribute

Note. Profiles represent qualitative patterns derived from the reviewed studies and should not be interpreted as diagnostic signatures. The evidence base column summarizes the number and broad design characteristics of the available studies and should not be interpreted as a formal grading of evidence certainty. Methodological appraisal of individual studies is reported in Supplementary Material S1. “Insufficient evidence” indicates that the available data do not permit a reliable characterization of the domain, whereas “Not specifically assessed” indicates that the available studies did not provide a sufficiently specific assessment of that domain. AD = Alzheimer’s disease; FTD = frontotemporal dementia; lvPPA = logopenic variant primary progressive aphasia; MCI = mild cognitive impairment; PD-NC = Parkinson’s disease with normal cognition; PD-MCI = Parkinson’s disease with MCI; svPPA = semantic variant primary progressive aphasia.

## Data Availability

No new data were generated in this study. All data supporting the findings of this systematic review are derived from the published studies included in the review.

## Supplementary Materials

The following supporting information can be downloaded at: https://www.mdpi.com/article/10.3390/brainsci16101067/s1. Supplementary Material S1. Methodological appraisal of included studies. Supplementary Material S2. Database Search Strategies.

## Abbreviations

The following abbreviations are used in this manuscript:

AD	Alzheimer’s disease
FTD	Frontotemporal dementia
lvPPA	Logopenic variant primary progressive aphasia
MCI	Mild cognitive impairment
PD-NC	Parkinson’s disease with normal cognition
PD-MCI	Parkinson’s disease with MCI
svPPA	Semantic variant primary progressive aphasia.
