# Supplementary material for: Numerical Cognition in Neurodegenerative Disorders: A Systematic Review of Patterns of Preservation and Impairment
Source: Brain Sci. 2026 Oct 4;16(10):1067. doi: 10.3390/brainsci16101067 (PMC13702540; doi:10.3390/brainsci16101067)
Supplement: Supplementary file 1 [file brainsci-16-01067-s001.zip › brainsci-4548854-supplementary.pdf]

Supplementary Material S1. Methodological appraisal of included studies

Study	Diagnosis	JBI checklist	Q1	Q2	Q3	Q4	Q5	Q6	Q7	Q8	Q9	Q10	Q11
Tegnér & Nybäck, 1990 [42]	AD	Analytical cross-sectional	Y	Y	Y	Y	N	N	NA	Y	—	—	—
Deloche et al., 1995 [16]	AD	Analytical cross-sectional	Y	Y	Y	Y	Y	N	Y	Y	—	—	—
Kessler & Kalbe, 1996 [43]	AD	Analytical cross-sectional	Y	Y	Y	Y	Y	N	Y	Y	—	—	—
Marterer et al., 1996 [48]	AD	Analytical cross-sectional	Y	Y	Y	Y	Y	N	Y	Y	—	—	—
Rosselli et al., 1998 [50]	AD	Analytical cross-sectional	Y	Y	Y	Y	Y	N	Y	Y	—	—	—
Hirono et al., 1998 [51]	AD	Analytical cross-sectional	Y	Y	Y	Y	Y	Y	Y	Y	—	—	—
Mantovan et al., 1999 [52]	AD	Analytical cross-sectional	Y	Y	Y	Y	Y	N	Y	Y	—	—	—
Carlomagno et al., 1999 [15]	AD	Analytical cross-sectional	Y	Y	Y	Y	Y	Y	Y	Y	—	—	—
Della Sala et al., 2000 [41]	AD	Analytical cross-sectional	Y	Y	Y	Y	Y	N	Y	Y	—	—	—
Crutch & Warrington, 2001 [23]	AD/FTD/svPPA	Analytical cross-sectional	Y	Y	Y	Y	Y	N	Y	Y	—	—	—
Halpern et al., 2003 [21]	AD/FTD/CBD	Analytical cross-sectional	Y	Y	Y	Y	Y	Y	Y	Y	—	—	—
Martin et al., 2003 [49]	AD	Analytical cross-sectional	Y	Y	Y	Y	Y	N	Y	Y	—	—	—
Duverne et al., 2003 [53]	AD	Analytical cross-sectional	Y	Y	Y	Y	Y	N	Y	Y	—	—	—
Martini et al., 2003 [40]	AD	Analytical cross-sectional	Y	Y	Y	Y	Y	N	Y	Y	—	—	—
Allen et al., 2005 [54]	AD	Analytical cross-sectional	Y	Y	Y	Y	Y	N	Y	Y	—	—	—
Zamarian, Semenza et al., 2007 [28]	AD/MCI	Analytical cross-sectional	Y	Y	Y	Y	Y	N	Y	Y	—	—	—
Cappelletti et al., 2012 [22]	AD/FTD/svPPA	Analytical cross-sectional	Y	Y	Y	Y	Y	N	Y	Y	—	—	—
Ting et al., 2016 [44]	AD	Analytical cross-sectional	Y	Y	Y	Y	Y	N	Y	Y	—	—	—

Study	Diagnosis	JBI checklist	Q1	Q2	Q3	Q4	Q5	Q6	Q7	Q8	Q9	Q10	Q11
Jurado & Rosselli, 2017 [26]	AD/MCI	Analytical cross-sectional	Y	Y	Y	Y	Y	N	Y	Y	—	—	—
Roquet et al., 2020 [19]	AD	Analytical cross-sectional	Y	Y	Y	Y	Y	N	Y	Y	—	—	—
Macoir et al., 2020 [27]	AD/MCI	Analytical cross-sectional	Y	Y	Y	Y	Y	N	Y	Y	—	—	—
Zamarian, Stadelmann et al., 2007 [25]	MCI	Analytical cross-sectional	Y	Y	Y	Y	Y	N	Y	Y	—	—	—
Benavides-Varela et al., 2015 [24]	MCI	Analytical cross-sectional	Y	Y	Y	Y	Y	Y	Y	Y	—	—	—
Julien et al., 2010 [58]	svPPA	Analytical cross-sectional	Y	Y	Y	Y	Y	N	Y	Y	—	—	—
Luzzi et al., 2013 [61]	svPPA	Analytical cross-sectional	Y	Y	Y	Y	Y	Y	Y	Y	—	—	—
Kas et al., 2011 [35]	PCA	Analytical cross-sectional	Y	Y	Y	Y	Y	Y	Y	Y	—	—	—
Zamarian et al., 2006 [37]	PD	Analytical cross-sectional	Y	Y	Y	Y	Y	N	Y	Y	—	—	—
Loenneker et al., 2024 [36]	PD	Analytical cross-sectional	Y	Y	Y	Y	Y	Y	Y	Y	—	—	—
Macoir et al., 2026 [38]	lvPPA	Analytical cross-sectional	Y	Y	Y	Y	Y	N	Y	Y	—	—	—
Grafman et al., 1989 [20]	AD	Case report	Y	Y	Y	Y	NA	NA	NA	Y	—	—	—
Cipolotti, 1995 [45]	AD	Case report	Y	Y	Y	Y	NA	NA	NA	Y	—	—	—
Noël & Seron, 1995 [46]	AD	Case report	Y	Y	Y	Y	NA	NA	NA	Y	—	—	—
Thioux et al., 1999 [47]	AD	Case report	Y	Y	Y	Y	NA	NA	NA	Y	—	—	—
Macoir et al., 2002 [18]	AD	Case report	Y	Y	Y	Y	NA	NA	NA	Y	—	—	—
McNeil & Burgess, 2002 [56]	AD	Case report	Y	Y	Y	Y	NA	NA	NA	Y	—	—	—
Diesfeldt, 1993 [31]	svPPA/SD	Case report	Y	Y	Y	Y	NA	NA	NA	Y	—	—	—
Cappelletti et al., 2001 [29]	svPPA/SD	Case report	Y	Y	Y	Y	NA	NA	NA	Y	—	—	—
Crutch & Warrington, 2002 [32]	svPPA/SD	Case report	Y	Y	Y	Y	NA	NA	NA	Y	—	—	—
Cappelletti et al., 2005 [30]	svPPA/SD	Case report	Y	Y	Y	Y	NA	NA	NA	Y	—	—	—
Jefferies et al., 2005 [33]	svPPA/SD	Case report	Y	Y	Y	Y	NA	NA	NA	Y	—	—	—
Zamarian et al., 2006 [59]	svPPA/SD	Case report	Y	Y	Y	Y	NA	NA	NA	Y	—	—	—
Papagno et al., 2013 [60]	svPPA/SD	Case report	Y	Y	Y	Y	NA	NA	NA	Y	—	—	—

Study	Diagnosis	JBI checklist	Q1	Q2	Q3	Q4	Q5	Q6	Q7	Q8	Q9	Q10	Q11
Delazer, Karner et al., 2006 [34]	PCA	Case report	Y	Y	Y	Y	NA	NA	NA	Y	—	—	—
Delazer et al., 2019 [55]	AD/MCI	Multiple single-case	Y	Y	Y	U	U	Y	Y	Y	Y	Y	—
Cheng et al., 2013 [57]	svPPA/SD	Case series	Y	Y	Y	U	U	Y	Y	Y	N	Y	—
Mendez et al., 2018 [39]	AD	Case series	Y	Y	Y	Y	Y	Y	Y	Y	Y	Y	—
Kaufmann et al., 2002 [17]	AD	Longitudinal group study	Y	Y	Y	Y	Y	NA	Y	Y	N	N	Y

Note. Ratings: Y = Yes; N = No; U = Unclear; NA = Not applicable; — = item not included in the relevant JBI checklist. No aggregate quality score or cross-design ranking was calculated.

Analytical cross-sectional checklist (Q1–Q8). Q1 = inclusion criteria clearly defined; Q2 = objective standard criteria used for the condition; Q3 = exposure/group status measured validly and reliably; Q4 = outcomes measured validly and reliably; Q5 = confounding factors identified; Q6 = strategies to deal with confounding stated; Q7 = appropriate statistical analysis; Q8 = subjects and setting described in detail.

Case report checklist (Q1–Q8). Q1 = demographic characteristics clearly described; Q2 = history clearly described and presented as a timeline; Q3 = current clinical condition clearly described; Q4 = diagnostic tests/assessment methods and results clearly described; Q5 = intervention/treatment clearly described; Q6 = post-intervention clinical condition clearly described; Q7 = adverse/unanticipated events identified and described; Q8 = takeaway lessons provided.

Case series checklist (Q1–Q10). Q1 = clear inclusion criteria; Q2 = condition measured in a standard, reliable way; Q3 = valid methods used for identification; Q4 = consecutive inclusion; Q5 = complete inclusion; Q6 = demographics reported; Q7 = clinical information reported; Q8 = outcomes/follow-up reported; Q9 = presenting site(s)/clinic(s) demographic information reported; Q10 = appropriate statistical analysis.

Cohort checklist (Q1–Q11). Q1 = groups similar and recruited from the same population; Q2 = exposures measured similarly; Q3 = exposure measured validly and reliably; Q4 = confounding factors identified; Q5 = strategies to deal with confounding stated; Q6 = participants free of the outcome at study start; Q7 = outcomes measured validly and reliably; Q8 = follow-up time reported and sufficient; Q9 = follow-up complete or reasons for loss described; Q10 = strategies used to address incomplete follow-up; Q11 = appropriate statistical analysis.

Supplementary Material S2. Database Search Strategies

The initial literature searches were conducted on April 20, 2026, in PubMed, PsycINFO (via Ovid), and Web of Science Core Collection, with no publication-date or language filters applied. The same database-specific search strategies were rerun on July 16, 2026, to identify potentially eligible records added since the initial searches. Eligibility was restricted to articles published in English or French during screening

Database	Search strategy
**PubMed**	(<br>"Alzheimer Disease"[Mesh]<br>OR "Aphasia, Primary Progressive"[Mesh]<br>OR "Frontotemporal Lobar Degeneration"[Mesh]<br>OR "Lewy Body Disease"[Mesh]<br>OR "Parkinson Disease"[Mesh]<br>OR "Mild Cognitive Impairment"[Mesh]<br>OR Alzheimer*[tiab]<br>OR dementia[tiab]<br>OR "mild cognitive impairment"[tiab]<br>OR "primary progressive aphasia"[tiab]<br>OR "frontotemporal dementia"[tiab]<br>OR "Lewy body disease"[tiab]<br>OR "Parkinson disease"[tiab]<br>OR "posterior cortical atrophy"[tiab]<br>)<br>AND<br>(<br>acalculi*[tiab]<br>OR dyscalculi*[tiab]<br>OR "number processing"[tiab]<br>OR "numerical processing"[tiab]<br>OR "numerical cognition"[tiab]<br>OR "number cognition"[tiab]<br>OR "number transcoding"[tiab]<br>OR transcoding[tiab]<br>OR "number comparison"[tiab]<br>OR "magnitude comparison"[tiab]<br>OR "number line"[tiab]<br>OR "mental number line"[tiab]<br>OR "numerical magnitude"[tiab]<br>OR "quantity processing"[tiab]<br>OR "arithmetic facts"[tiab]<br>)
**PsycINFO (Ovid)**	1. (Alzheimer* or dementia or "mild cognitive impairment" or "primary progressive aphasia" or "frontotemporal dementia" or "Lewy body disease" or Parkinson* or "posterior cortical atrophy").ti,ab.<br><br>2. (acalculi* or dyscalculi* or "number processing" or "numerical processing" or "numerical cognition" or "number cognition" or "number transcoding" or transcoding or "number comparison" or "magnitude comparison" or "number line" or "mental number line" or "numerical magnitude" or "quantity processing" or "arithmetic facts").ti,ab.<br><br>3. 1 and 2
**Web of Science Core Collection**	TS=(<br>"Alzheimer disease"<br>OR dementia<br>OR "mild cognitive impairment"<br>OR "primary progressive aphasia"<br>OR "frontotemporal dementia"

	<pre> OR "Lewy body disease"<br>OR "Parkinson disease"<br>OR "posterior cortical atrophy"<br>)<br>AND<br>TS=(<br>acalculia<br>OR dyscalculia<br>OR "number processing"<br>OR "numerical cognition"<br>OR "number cognition"<br>OR transcoding<br>OR "number comparison"<br>OR "magnitude comparison"<br>OR "number line"<br>OR "mental number line"<br>)</pre>
